# The Connection Between Stress and Immune Status in Pigs: A First Salivary Analytical Panel for Disease Differentiation

**DOI:** 10.3389/fvets.2022.881435

**Published:** 2022-06-16

**Authors:** J. Sánchez, M. Matas, F. J. Ibáñez-López, I. Hernández, J. Sotillo, A. M. Gutiérrez

**Affiliations:** ^1^BioVetMed Research Group, Department of Animal Medicine and Surgery, Veterinary School, CEIR Campus Mare Nostrum (CMN), University of Murcia, Murcia, Spain; ^2^Cefu SA, Murcia, Spain; ^3^Statistical Support Service (SAE), Scientific and Technological Research Area (ACTI), CEIR Campus Mare Nostrum (CMN), University of Murcia, Murcia, Spain

**Keywords:** principal component analysis, disease discrimination, salivary analytics, pig, field study

## Abstract

This paper analyzes the association between stress and immune response activations in different diseases, based on the salivary analytics. Moreover, a first attempt to discriminate between diseases was performed by principal component analysis. The salivary analytics consisted of the measurement of psychosocial stress (cortisol and salivary alpha-amylase) indicators, innate (acute phase proteins: C-reactive protein and haptoglobin), and adaptive immune (adenosine deaminase, Cu and Zn) markers and oxidative stress parameters (antioxidant capacity and oxidative status). A total of 107 commercial growing pigs in the field were divided into six groups according to the signs of disease after proper veterinary clinical examination, especially, healthy pigs, pigs with rectal prolapse, tail-biting lesions, diarrhea, lameness, or dyspnea. Associations between stress and immune markers were observed with different intensities. High associations (*r* = 0.61) were observed between oxidative stress markers and adaptive immune markers. On the other hand, moderate associations (*r* = 0.31–0.48) between psychosocial stress markers with both innate and adaptive immune markers were observed. All pathological conditions showed statistically significant differences in at least 4 out of the 11 salivary markers studied, with no individual marker dysregulated in all the diseases. Moreover, each disease condition showed differences in the degree of activation of the analyzed systems which could be used to create different salivary profiles. A total of two dimensions were selected through the principal component analysis to explain the 48.3% of the variance of our data. Lameness and rectal prolapse were the two pathological conditions most distant from the healthy condition followed by dyspnea. Tail-biting lesions and diarrhea were also far from the other diseases but near to healthy animals. There is still room for improvements, but these preliminary results displayed a great potential for disease detection and characterization using salivary biomarkers profiling in the near future.

## Introduction

The harmful effect of many stressors on immunity has been defined before (1–5) and consists of a minor reaction, or an acute clinical reaction which is only apparent for a short duration (1). Moreover, it has been described that one potential indication of decreased welfare in pigs is a greater prevalence of disease because of compromised immunity in “stressed” animals (2). However, long-term effects of stress, such as immunosuppressive or immunomodulatory function, over immunity seem to be linked to a chronic condition. This chronic stress could produce the changes in innate immunity in which leukocytes transmigrate through the endothelial lining and infiltrate inflamed site and thus lead to a decrease in blood leukocyte numbers (3). On the other hand, to study the effects of stress on adaptive immune responses, lymphocyte proliferation status has often been employed (4) with the suppression of lymphocyte proliferation in repeated stress models (5).

Chronic psychological stress appears to accelerate biological aging, of which oxidative damage is an important potential mediator (6). Free radicals protect against invading microorganisms and can cause tissue damage during inflammation as well (7). Evidence of oxidative stress has been observed in several infectious diseases in farm animals (8).

Likewise, inflammation is considered as a mechanism of innate immunity that must be tightly controlled to be beneficial for the host (9). Cytokines released upon the activation of the immune system stimulate the hypothalamic–pituitary–adrenal (HPA) axis, increase the peripheral levels of glucocorticoids (10), and consequently produce acute stress response.

The connection between stress and disease was well documented (2). Most of the studies have reported the association between abnormal animal behaviors, such as excessive aggressive interactions or vocalizations or long periods without any exploratory behavior, and stress, and the increased disease risk or even the variation in the level of immune markers after the experimental application of different stressors (11–13). Fewer studies evaluated the variation of immune markers in stressful field conditions in pigs, and even a smaller number of studies have compared the behavior of stress markers in field disease condition.

Saliva is the biological fluid commonly used for the determination of stress biomarkers such as cortisol (14) and immune titers (15). In humans, it has been reported that sympathetic adrenal medullary (SAM) system is activated after both psychologically and physically stressful task, with an increase in the levels of salivary alpha-amylase levels, whereas the HPA axis is mainly activated in response to physical stressors with an increase in the levels of cortisol in saliva samples (16). In pigs, it has been described that the different axes involved in the stress response are differently activated, depending on the stressor (11). Additionally, both physical and social stressors have an additive effect (17). As a result, it seems that for a complete stress response evaluation, the SAM system and the HPA axis should be checked parallelly.

The monitoring of a pig's health using saliva samples or oral fluids has been of strong interest in the last decade and has been mainly focusing on the detection of specific pathogens, or the evaluation of the acute phase reaction (18). However, the use of more than one acute phase protein for health status evaluation has been widely recommended due to the high variation in response against different homeostasis disturbances of each protein (19). The superiority of saliva over serum has been recently explained for disease detection in pigs using a set of four biomarkers, including two acute phase proteins, an immune marker, and the total antioxidant capacity (20).

Saliva offers several strengths as an analytical fluid since saliva samples can be obtained by personnel with minimal training. This could facilitate rapid data collection for regional or national disease control or eradication programs (21). Furthermore, the collection procedure is efficient and low cost. Moreover, saliva sampling could eliminate the risk of moving viruses between sites if performed by barn personnel, in comparison with blood samples, which facilitates the collection of large number of diagnostic samples (22).

The establishment of high sanitary and welfare standards is a major concern of consumers, producers, and regulators nowadays in the European Community and is one of the three general objectives of the EU about food safety policy (23). Consequently, there is a global interest in developing cost-effective and sustainable animal production models based on the high sanitary and welfare standards. The monitoring of the principal components of the stress response, the occurrence of oxidative stress, and the health status of animals at farm level should be of help to fulfill these demands.

Machine learning algorithms could produce a model which will differentiate among the output labels based on the input dataset in laboratory conditions (24). The application of machine learning tools in farm animals has been accompanied by the development of precision livestock farming tools in the recent years and could be of help for the identification of key markers associated with a specific health status. This approach has been previously and recently used for the detection of factors associated with porcine reproductive and respiratory syndrome virus (PRRSV) outbreaks (25) or to study the behavioral traits for animal well-being assessment (26). One of the goals of precision livestock farming is the early detection of illness or physiological status at the farm level, so there is a niche for machine learning approaches within animal production (27).

This study aims to differentiate between common multifactorial porcine diseases, for the first time, using a salivary analytical panel, and to analyze the connection between stress and immune status biomarkers in field conditions. Our hypotheses were that: (i) the degree of an alteration in the diverse physiological host responses is not identical in all diseases and, if properly measured, could be used for disease discrimination; and (ii) the connection between immune and stress markers could be analyzed in saliva samples of pigs and an influence of disease condition could be expected.

## Materials and Methods

The ARRIVE guidelines have been considered for the design, analysis, and reporting of scientific research in this study (28).

### Animals and Housing Conditions

All procedures involving animals were approved by the Ethics Committee of the University of Murcia and followed the European (29) and the Spanish regulation (30) on the protection of animals used for scientific purposes.

A total of four commercial farms from the southeast of Spain were selected from the same commercial company to obtain data over different pathologies and environments. The vaccination program was the same for all farms and included vaccination against enzootic pneumonia, porcine circovirus, and Aujeszky's disease at the age of 7 and 28 days, at the age of 28 days, and 11 and 14 weeks of life. Pigs were housed in pen groups with 0.65 m^2^/pig following the official standards (31) with *ad libitum* access to balanced dry food and water.

The sample size needed for both objectives of the study, correlation analysis, and disease detection was estimated according to the guidelines of the Ethical Committee for Animal Research from the University of Murcia (approval number 561/2019, 14 November 2019), using specific statistical tools (G^*^Power 3.1 software) (32, 33). For disease detection with an estimated effect size of 1.51 [the highest effect size recently reported for disease detection (20)], an expected statistical power of 90% and an alpha error level of 0.05, the analysis suggested a minimal sample size of nine animals per group. Moreover, for an estimated minimal correlation coefficient of 0.3 (minimal size that could be interpreted as “low correlated”), an expected statistical power of 90% and an alpha error level of 0.05, the total samples size suggested for the analysis was 88.

The inclusion criteria were a maximum of 15 and a minimum of 10 for pigs without any clinical signs of disease, and for animals with any clinical signs of disease from each farm after veterinary clinical examination of commercial pigs at the finishing stage (between 120 and 180 days of age). Pigs without any clinical sign of disease during veterinary clinical examination at farm (*n* = 40) were classified as healthy. After recording all the symptoms from animals suffering from any pathology, five groups of animals with similar clinical signs of disease were formed: tail-biting lesions (*n* = 13), rectal prolapse (*n* = 13), diarrhea (*n* = 13), lameness (*n* = 14), and dyspnea (*n* = 14). Each pathological group was integrated with diseased animals from the same farm with minor exceptions. A total of three pigs with rectal prolapse were sampled from the farm with tail-biting problems. Moreover, a pig with tail-biting lesions was sampled during the visit to the farm with lameness problems (for more individual information, refer to raw data document). A total of 107 animals were selected for the study in five different batches, one per outbreak or pathology, between February and October 2020, especially 14 and 21 February, 17 June, 11 September, and 20 October.

For animal selection in each diseased group, the following criteria were established. The tail-biting animals were selected when acute lesion was observed. The group was composed of nine animals with minor acute wound and four pigs with major acute wound, according to the previously defined tail scoring system (34). All animals suffering from rectal prolapse showed an evident prolapse with different degrees of mucosa ulceration. The diarrhea outbreak was characterized by profuse mucohemorrhagic diarrhea and loss of body condition in all the selected animals, caused by *Brachyspira hyodysenteriae* according to qPCR analysis from the feces of diseased pigs. Lameness was defined as animals with deformans arthritis, evident swollen joints, and/or external abscess. The etiological agent of the dyspnea was *Actinobacillus pleuropneumoniae (App)* according to the postmortem examinations of dead animals. The *App* outbreak was characterized by animals with dyspnea, prostration, lack of appetite, lack of growth, and cough.

Animals from two of the farms were PIC x Danbred (57.9% of the animals), and animals from one farm were F1 Danbred (23.3% of the animals), whereas animals from the last farm were Duroc x Danbred (18.7% of the animals). Furthermore, the gender distribution of the animals included in the study was 35.5% female and 64.5% male (25 and 41.8% of females in healthy and diseased animals, respectively).

### Sampling Procedure

Saliva samples were collected individually, at the same time of the day (between 11 and 13 h) by the same personnel, without animal restrain, using 1 cm x 1 cm x 1 cm sponges clipped to a thin metal rod. Pigs were allowed to chew the sponge for 1–2 min. Afterward, sponges were included in specifically designed tubes for saliva collection (Salivette tubes, Sarstedt, Nümbrecht, Germany) and stored in boxes with cold accumulators until transported to the laboratory within 1.5 h after collection. Saliva collection tubes were centrifuged at 3,000 g for 10 min to obtain the clear whole saliva from the sponges and remove food or cell debris. Saliva samples were stored at −80°C until analysis.

### Immune Status Biomarker Quantification

For the monitoring of innate immunity system, two acute phase proteins were measured, especially C-reactive protein (CRP) and haptoglobin (Hp), whereas the evaluation of the adaptive immune system was performed by quantifying the levels of adenosine deaminase (ADA), Cu and Zn levels.

In-house non-competitive sandwich time-resolved immunofluorometric assays, developed and validated previously for the optimal quantification of CRP (35) and Hp (36) in porcine saliva samples, were used. The assay fluorometric signal was quantified in a multilabel counter (Victor 1420, PerkinElmer, Turku, Finland). The overall coefficients of variation were below 10 and 11% for CRP and Hp measurements, respectively, and the limits of detection of the assays were around 0.7–0.5 ng/ml (35, 36).

The enzyme activity levels of total ADA were quantified using a microplate adaptation of a commercial automatized assay (BioSystems S.A., Barcelona, Spain) that had previously been validated for porcine saliva measurements (37). The assay consisted of the reaction of 50 μl of a 1:16 saliva dilution with 200 μl of ADA reagent. The absorbance was monitored for 3 min at 340 nm, and the ADA activity level was obtained by calculating the maximal decrease in absorbance per minute (U/L in the applied sample = Δabs/min ^*^ 3,333). Values were given in U/L in the diluted saliva. The intra-assay coefficient of variation of the assay was lower than 6%, and the limit of detection was 9.3 U/L (37).

For the measurements of Cu and Zn levels, saliva samples were subjected to acid digestion as reported previously (38), followed by atomic absorption spectrometric quantifications (using a Varian model SpectrAA 55B spectrometer, Palo Alto, CA, USA). Certified standard solutions for Cu and Zn (1g/L) (Agilent Technologies Spain, Madrid, Spain) were used for calibration. The results were expressed as μg/ml.

### Oxidative Stress Biomarker Quantifications

The quantification of the antioxidant status in saliva samples of pigs was performed through the ferric reducing antioxidant power assay (39) previously optimized and validated for saliva samples in pigs (40). The optimized assay consisted of the reaction of 36 μl of saliva sample with 270 μl of ferric reducing power reagent. The antioxidant activity was calculated as the interpolation of measured absorbance in a calibration curve performed with Trolox (Sigma Aldrich, Darmstadt, Germany) using a linear regression curve fit based on blank corrected. The coefficients of variation were below 11%, and the limit of detection was 0.80 μm Trolox equivalents/L.

For the measurement of the oxidant status, a commercially available assay (PierceTM Quantitative Peroxide assay, ThermoScientific) was optimized and validated for its use in porcine saliva samples. The assay was optimized by testing different samples/working buffer ratios in different saliva dilutions. The optimized assay consisted of the reaction of 150 μl of saliva sample in a 1:2 dilution with 70 μl of working buffer. The calibration curve was constructed with peroxide in a range of 31.25–0.97 μm peroxidase equivalents. The mean coefficients of variation were 8.44% for the intra-assay precision and 10.8% for the inter-assay precision. The detection limit was 0.5 μm peroxidase equivalents/L.

The oxidative stress index (OSI) was calculated as the ratio TOS/TAC according to the previous studies (41).

### Psychosocial Stress Biomarkers Quantification

The monitoring of the HPA axis and the SAM system was evaluated by the measurements of the levels of cortisol and salivary alpha-amylase, respectively.

The cortisol content in the salivary samples was measured using an optimized commercial competitive ELISA (Extended range high sensitivity salivary cortisol Enzyme immunoassay kit, Salimetrics, USA). Cortisol levels in μg/dl were calculated according to the manufacturer's instructions by interpolation of the percentage bound in a 4-parameter nonlinear regression curve (standard curve range 0.012 μg/dl−3 μg/dl).

Salivary alpha-amylase was quantified using an optimized commercial kinetic enzyme assay (Salivary Alpha-amylase Kinetic Enzyme Assay Kit, Salimetrics, USA). The optimization consisted of the reaction of 8 μl of saliva with 320 μl of amylase substrate during 5 min at 37°C. The levels of alpha-amylase activity were calculated in U/L according to the manufacturer's instructions.

### Total Protein Content Determination

The total protein content of all saliva samples was determined according to the standard protocols (42). Saliva samples were diluted (1:40) for its proper quantification using a calibration curve with a 5–100 ng/ml concentration range of bovine serum albumin (Sigma Aldrich, Darmstadt, Germany).

### Statistical Analysis

All groups of animals were tested for normality and homoscedasticity prior analysis, using Shapiro–Wilk and Fligner–Killeen test, respectively. A total of two different approaches were performed for the search for statistically significant differences between analytes and to investigate the association between the different variables studied. The first approach included a “global” analysis that included all the studied animals. The second “individual” approach was performed by preparing different datasets including groups of healthy and diseased pigs coming from the same farm, since each disease appeared predominantly in one farm.

For the overall comparisons between the biomarkers from different groups of animals and the whole group of healthy pigs (healthy animals from four different commercial farms), Welch ANOVA test followed by Dunnett's T3 multiple comparisons test was used for data not normally distributed without homoscedasticity. Kruskal–Wallis test followed by Dunn's multiple comparisons test was applied when homoscedasticity was present in not normally distributed data.

To analyze the magnitude of the effect of each biomarker alteration in every disease, the Cohen's *d* for independent groups was individually calculated after proper statistical comparison (Supplementary Table 1). In brief, a *t*-test with Welch correction was used for data not normally distributed without homoscedasticity, nonparametric Mann–Whitney test was used for data not normally distributed but with homoscedasticity, and *t*-test was used for data normally distributed with homoscedasticity.

Principal component analysis (PCA) was used to identify possible underlying patterns of the data from the salivary biomarker levels in animals with different pathological conditions. First, the analysis reduced the number of dimensions in a dataset by feature extraction which uses the original variables to construct a new set of variables [named principal components (PCs), or dimensions (Dims)]. Afterward, the PCs or Dims 1 and 2, with a cumulative proportion of variance of 57.78%, were projected in a graph that showed the distance between the variance of the different diseased.

The global correlation between the different variables was performed using Spearman's correlation test since data did not pass the normal distribution criteria. To look for any possible statistically significant difference between the correlations of immune and stress biomarkers among the different pathological conditions that were studied, the individual Spearman's correlation coefficients were compared, and the size effect was calculated by the Cohen's *q* for correlated measurements. It is necessary to indicate that the sample size for the individual correlation analysis is not adequate for correlation values of *r* < 0.54. Therefore, results out of these criteria should be interpreted with caution.

All statistics were performed using R software version 4.0.3. The level of significance was set at *p* < 0.05.

## Results

### Disease Differentiation

**Figure 1:**
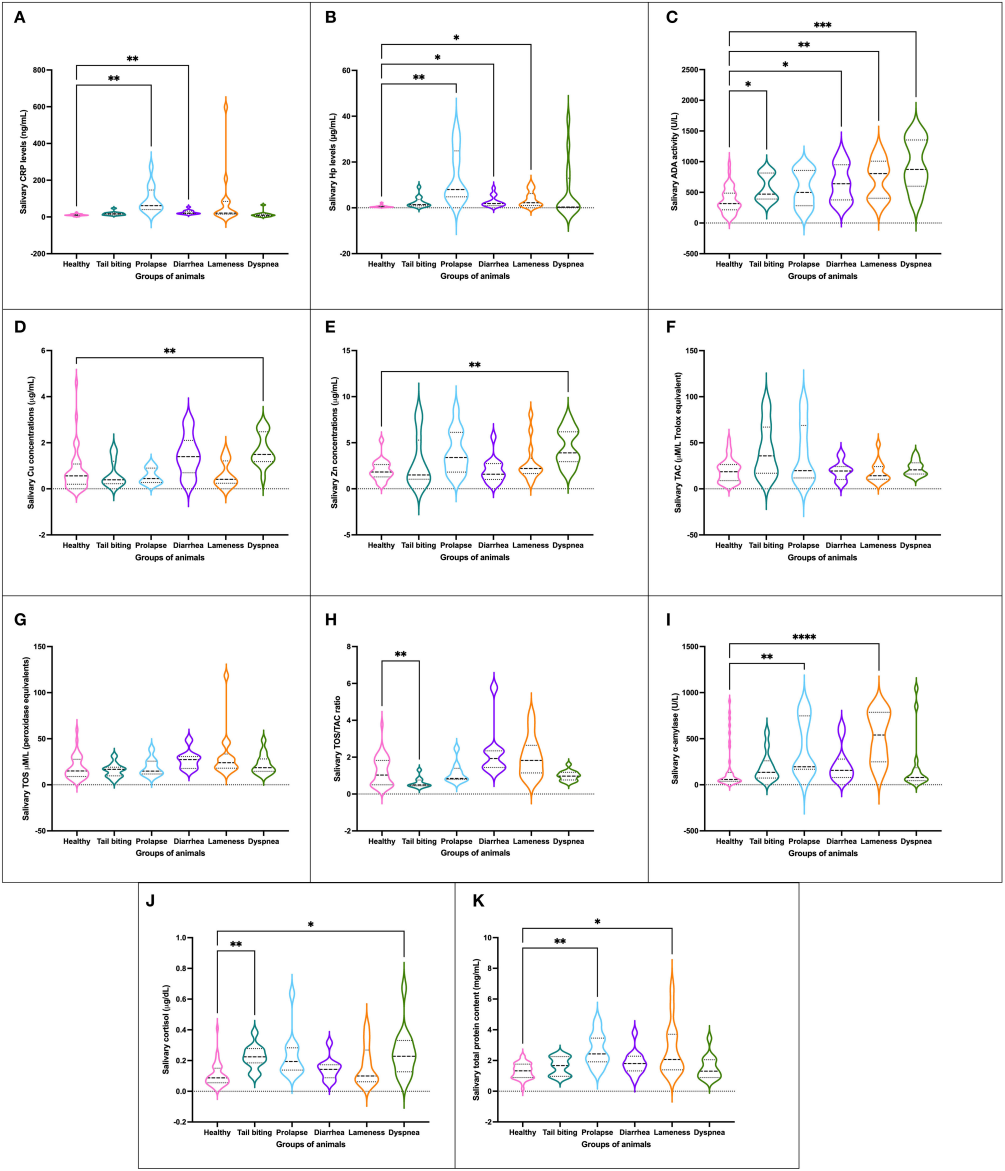
Concentration of salivary analytes studied [CRP **(A)**, Hp **(B)**, ADA **(C)**, Cu **(D)**, Zn **(E)**, TAC **(F)**, TOS **(G)**, ratio TOS/TAC **(H)**, salivary alpha-amylase **(I)**, cortisol **(J)** and TP **(K)**] in healthy pigs (*n* = 40) and animals suffering from a pathological condition (tail-biting lesions *n* = 13, rectal prolapse *n* = 13, diarrhea *n* = 13, lameness *n* = 14, or dyspnea *n* = 14). Graph showing the distribution of the population (depending on whether the plot is widening or narrowing), the median (central horizontal line), 25th and 75th percentiles (non-central horizontal lines within the plot), maximum and minimum (edges of the figure). Statistical differences are indicated by ^*^, ^**^, ^***^, and ^****^ for *p* < 0.05, *p* < 0.01, *p* < 0.001, and *p* < 0.0001, respectively. CRP, C-reactive protein; Hp, haptoglobin; ADA, adenosine deaminase; TAC, total antioxidant capacity; TOS, total oxidant status; TP, total protein.

An alteration in at least one of the innate (CRP and Hp) or adaptive immune markers (ADA, Zn, and Cu) quantified was presented in the five pathologies studied when compared to the whole group of healthy animals. Statistically significant increases in CRP and/or Hp levels were observed in pigs with prolapse, diarrhea, and lameness (Figures 1A,B). For ADA activity levels, all pathological conditions but prolapse showed statistically significant higher values than those observed for healthy animals (Figure 1C). Moreover, pigs suffering from dyspnea showed statistically significantly higher levels of Cu (Figure 1D) and Zn (Figure 1E) as well.

No statistically significant changes in the levels of TAC, TOS, or OSI were observed in any of the pathological conditions studied with the exception of the group of animals with tail-biting lesions, in which a decrease in OSI was observed in comparison with healthy pigs (Figures 1F–H). However, a trend of increased OSI was shown in the group of animals with diarrhea and lameness in comparison with healthy pigs.

A total of four groups of pigs with pathological conditions showed variations in the concentration of psychosocial stress markers in comparison with healthy pigs. Two groups of animals, especially those with rectal prolapse and lameness, and other two groups of pigs, those suffering from tail-biting lesions and dyspnea, showed statistically significant increases in the levels of alpha-amylase (Figure 1I) and cortisol (Figure 1J), respectively, in comparison with healthy pigs.

The mean total protein concentration in saliva samples of the group of healthy animals was statistically significantly lower than the average levels observed in the groups of pigs with rectal prolapse and lameness (Figure 1K).

Different effect sizes were observed in the alteration of salivary biomarkers in each specific disease when analyzed in the individual approach (Table 1). Some biomarkers behave completely different depending on the disease analyzed (6 out of 11); however, other biomarkers showed similar alterations in two or more pathological conditions (4 out of 11).

**Table 1:** Statistically significant differences (asterisks) between healthy animals (*n* = 10) and animals suffering from one pathology (tail-biting lesions *n* = 13, rectal prolapse *n* = 13, diarrhea *n* = 13, lameness *n* = 14, or dyspnea *n* = 14) from the same farm.

**Variable**	**Tail biting**	**Prolapse**	**Diarrhea**	**Lameness**	**Dyspnea**
CRP	* (0.91)	** (1.48)	*** (0.72)		
Hp	** (1.16)	** (1.46)	* (0.48)	** (1.29)	
ADA	* (0.43)	** (0.57)		* (0.53)	**** (2.15)
Cu		** (0.94)		* (0.49)	**** (2.82)
Zn		** (0.91)			**** (1.74)
TAC					
TOS					** (1.17)
Ratio TOS/TAC					
Amylase	* (0.72)		* (0.77)	**** (2.42)	
Cortisol	* (0.43)	* (1.37)	* (0.45)		** (1.29)
PT		*** (1.75)		** (1.06)	

*The size effect is represented as the coefficient Cohen's d in brackets. For more information about exact p-values and statistical test used refer to Supplementary Table 1. CRP, C-reactive protein; Hp, haptoglobin; ADA, adenosine deaminase; TAC, total antioxidant capacity; TOS, total oxidant status; TP, total protein*.

^*^ p < 0.05.

^**^ p < 0.01.

^***^ p < 0.001.

^****^ p < 0.0001.

**Figure 2:**
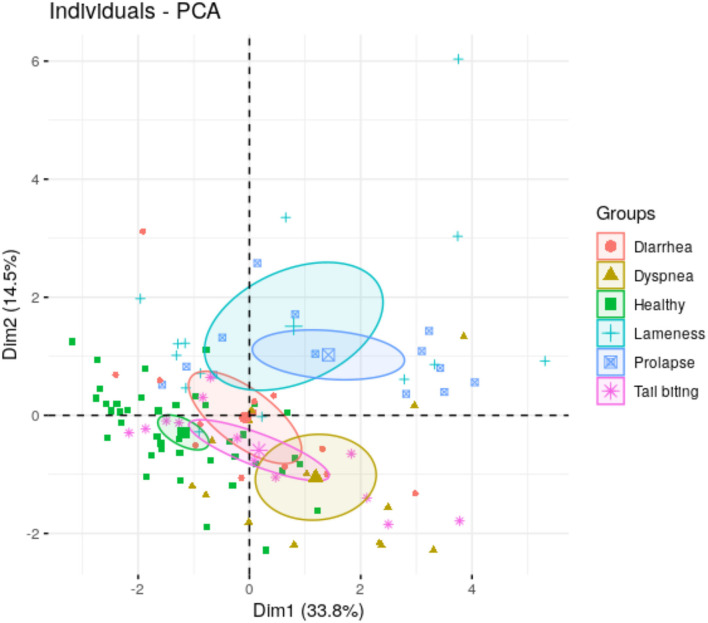
Principal components 1 (Dim 1) and 2 (Dim 2) of salivary biomarkers data form animals under different health conditions (healthy, rectal prolapse, tail-biting lesions, diarrhea, lameness, or dyspnea). In brackets, the percentage of total variance explained the principal component.

The preliminary PCA analysis showed the maximum variation of our data in each pathological condition using two dimensions (Dim 1 and Dim 2) (Figure 2). Dim 1 and Dim 2 appeared negative in healthy conditions, whereas in the group of lameness and prolapse, both dimensions appeared positive. In animals with dyspnea, Dim 1 appeared positive and Dim 2 was negative. Dim 1 appeared neutral in animals with tail-biting lesions, in which Dim 2 was negative. No influence of Dim 1 or Dim 2 was observed in the group of animals with diarrhea. The contribution of each biomarker or variable to Dim 1 and Dim 2 could be observed in Supplementary Figure 1.

### Associations Between the Levels of Immune and Stress Markers

**Figure 3:**
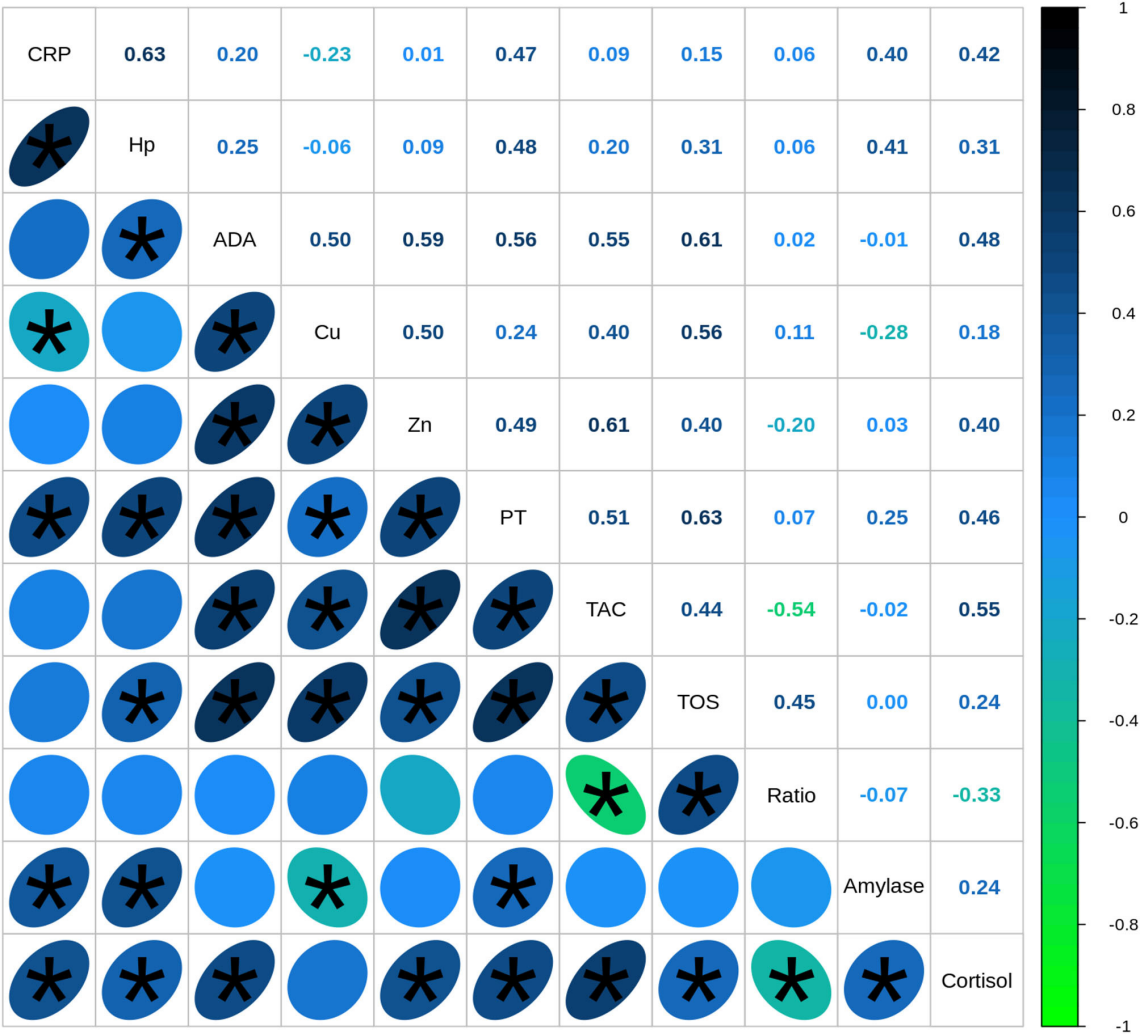
Spearman's correlation coefficients between biomarkers quantified in saliva of healthy (*n* = 40) and diseased (*n* = 67) pigs. CRP, C-reactive protein; Hp, haptoglobin; ADA, adenosine deaminase; TAC, total antioxidant capacity; TOS, total oxidant status; TP, total protein. Correlations with statistical significance are indicated by *.

The correlation analysis between all the studied biomarkers showed a total of 29 out of 55 associations between variables with statistical significance, and medium (0.3 < *r* < 0.5) or high effect (*r* > 0.5) (Figure 3). The highest statistically significant associations were observed between immune markers and oxidative stress markers, especially between ADA and Zn with TOS and TAC, respectively (*r* = 0.61). TAC levels were associated with ADA (*r* = 0.55) and Cu levels (*r* = 0.40) as well, whereas TOS values were also correlated with Cu (*r* = 0.56), Zn (*r* = 0.40), and Hp (*r* = 0.31). Regarding the correlations between immune and psychosocial stress markers, the highest statistically significant correlations were observed between cortisol and ADA levels (*r* = 0.48) followed by cortisol and CRP (*r* = 0.42), Zn (*r* = 0.40), and Hp (*r* = 0.31). Alpha-amylase levels were positively associated with Hp levels (*r* = 0.41) and CRP concentrations (*r* = 0.40) as well and presented a weak negative association with Cu levels (*r* = −0.28).

Moreover, when the correlations between biomarkers were analyzed individually in each disease group—including animals with a specific pathological conditions and healthy animals from the same origin—a total of 35 associations out of 125 fulfill the criteria of showing a correlation coefficient *r* > 0.54 and could be properly explained. The rest of the associations should be interpreted with caution. Several statistically significant differences were observed in the associations between the different pathological conditions analyzed (Table 2). First, statistically significant positive correlations were detected between ADA and TOS and Zn and TOS concentrations in all the diseases with similar correlation coefficients. Moreover, cortisol was associated with at least one immune marker in all diseases but with notably variations in the correlation coefficient between conditions whereas alpha-amylase mainly showed weak associations with immune markers in lameness and, in a less extend, in the tail-biting group of animals.

**Table 2:** Spearman's correlation coefficients between immune and stress biomarkers in animals suffering from a pathological condition (tail-biting lesions *n* = 13, rectal prolapse *n* = 13, diarrhea *n* = 13, lameness *n* = 14, or dyspnea *n* = 14) including a healthy group of animals (*n* = 10) from the same farm.

**Pair of analytes**	**Tail biting**	**Prolapse**	**Diarrhea**	**Lameness**	**Dyspnea**
**Immune vs. psychosocial stress markers**					
CRP-Amylase	0.32	−0.1^a^	0.26	0.53^b^	0.43
CRP-Cortisol	0.3	0.55	0.46	0.73^a^	0.23^b^
Hp-Amylase	0.55	0.01^a^	0.1	0.65^b^	0.27
Hp-Cortisol	0.65^a^	0.79^a^	0.37	0.49	−0.07^b^
ADA-Amylase	0.29	0.03	−0.24	0.37^a^	−0.25^b^
ADA-Cortisol	0.39^a^	0.80^b^	0.28^a^	0.49	0.84^b^
Cu-Amylase	0.14	−0.12	−0.27	−0.52	−0.35
Cu-Cortisol	0.3	0.74^a^	0.55	−0.02^b^	0.64^a^
Zn-Amylase	0.12	0.01	−0.31	0.15	−0.33
Zn-Cortisol	0.19	0.4	0.16	0.36	0.61
**Immune vs. oxidative stress markers**					
CRP-TAC	−0.06	0.05	0.1	0.31	−0.04
CRP-TOS	0.14	0.45	0.29	0.29	0.13
CRP-Ratio	0.01	0.48	0.24	0.07	0.14
Hp-TAC	0.36	0.48	0.4	0.31	0.12
Hp-TOS	0.56	0.66^a^	0.53	0.4	0.02^b^
Hp-Ratio	−0.12	0.15	0.08	0.11	−0.16
ADA-TAC	0.79	0.75	0.59	0.41	0.6
ADA-TOS	0.8	0.69	0.55	0.46	0.73
ADA-Ratio	−0.16	−0.08	−0.22	0.07	0.31
Cu-TAC	0.82^a, b^	0.23^c^	0.79^b^	0.54	0.39^b, c^
Cu-TOS	0.52	0.49	0.57	0.33	0.66
Cu-Ratio	−0.52^a^	0.32^b, c^	−0.45^a^	−0.19^a, c^	0.49^b^
Zn-TAC	0.75	0.37	0.75	0.77	0.64
Zn-TOS	0.5	0.58	0.63	0.65	0.63
Zn-Ratio	−0.37	0.26^a^	−0.48^b^	−0.12	0.21^a^

*CRP, C-reactive protein; Hp, haptoglobin; ADA, adenosine deaminase; TAC, total antioxidant capacity; TOS, total oxidant status; TP, total protein*.

*^a−c^Correlations with different superscripts within rows differ significantly (p < 0.005)*.

Regarding the oxidative stress markers TOS and TAC, the analysis showed that the levels of both markers were highly correlated with adaptive immune markers in all diseases without wide differences in the correlation coefficients. The statistically significant variations between the correlation coefficients were observed in the comparison between Cu and TAC levels within four of the animal groups and in the Hp and TOS levels in the group of prolapse and dyspnea.

## Discussion

First, this study analyzed the possible differentiation of common multifactorial porcine pathological conditions using a set of salivary biomarkers (psychosocial stress, oxidative stress, acute phase reaction, and adaptive immune markers). A total of five different common pathologies in commercial porcine growing were selected: tail-biting lesions, rectal prolapse, diarrhea, lameness, and dyspnea. The pathogenesis and etiology of these five common pathological conditions, which cause important loses in porcine production, are diverse and multifactorial. It has been reported that tail-biting lesions are highly related to stressful housing conditions that produce excessive motivation for biting (43). However, tail-biting lesions are also suggested to be an indication of serious stress experienced by the victims (44). In our study, animals suffering from tail-biting lesions showed high salivary levels of cortisol and amylase which agree to an activation of the HPA axis and the SAM system activity due to stress (45). Several external factors influence the prevalence of tail-biting lesions, including barren rearing environment, the lack of rooting materials, high socking densities, and herd sizes, lack off deeding space, or even conditions with high temperatures and low ventilation (46), which could also produce the development of distress response and immune activation (47). Moreover, suboptimal health has been also defined as a risk factor for tail biting (48). In this study, an alteration of health status was detected by an increase in salivary acute phase proteins and ADA activity levels as reported before, together with the psychological stress reaction, in animals with tail-biting lesions (20). Moreover, it has been reported that pigs with tail-biting lesions experienced an intense inflammatory condition leading to an acute phase response which is associated with the formation of carcass abscesses when examined at abattoirs (49), which also agree with our high level of immune markers.

The exact mechanisms that explain the development of rectal prolapse in growing pigs are not understood, but the fundamental cause is an increase in abdominal pressure resulting from many clinical conditions (50). High stocking densities, wet housing conditions, cold weather, mycotoxins, or even disbalance diet have been postulated to be causal or contributory factors for the development of rectal prolapse (51). The multifactorial component of this disease has been pointed out in our group of animals with rectal prolapse, since an activation of the hypothalamic–pituitary–adrenal (HPA) axis, detected by increasing cortisol and amylase, which showed that animals were subjected to stressful conditions, together with significant increases in innate and adaptive immune markers, which showed an intense inflammatory and immune activation, was observed. Additionally, both the HPA and the immune activation could be a reflection of the pain experienced by the rectal prolapse itself (52).

Swine dysentery is a severe enteric disease in pigs that produce a severe diarrhea. The etiology of swine dysentery is *Brachyspira hyodysenteriae*. However, several factors have been associated with disease expression including stressful management procedures, other environmental aspects such as stoking density, the presence of organic matter or the environmental temperature, and biosecurity and husbandry factors (53). An increase in acute phase proteins has been described at the onset of clinical disease of swine dysentery (54) in concordance with our results of the saliva samples. Moreover, we have also observed an increase in adaptive immune markers—ADA, Cu, and Zn—that could be related to the development of a specific immune response to outer membrane antigens (55). The stress condition related to the development of the disease was observed in our animals with an increase in the levels of stress biomarkers related to the HPA axis and the autonomic (sympathetic) nervous system.

Lameness in pigs represents a serious welfare problem but also because it has a detrimental impact on profitability (56). Moreover, lameness reduces the numbers of finisher pigs reaching the factory (57), so early detection or even good prevention would be the most important issues to consider. One of the major factors that influence lameness in commercial pig farms is the housing type followed by floor type. Specifically, fully slatted concrete flooring, minimal space allowances, and competitive feeding systems are associated with an increased risk of lameness (58). All those factors that seem to increase the risk of lameness are associated with poor welfare and health status conditions (56, 57). The salivary analysis in the lameness group in this study showed an activation of the autonomic nervous system and the immune system that could be associated with pain and tissue damage (52), which could be interpreted as the lack of optimal welfare and health status as well.

*Actinobacillus pleuropneumoniae* produce pleuropneumonia in pigs with dyspnea as the main clinical alteration (heavy breathing or open-mouth breathing) together with coughing or even sudden death (59). Experimental infections showed a rapid increase in several acute phase proteins until 15 days post-infection (60). The respiratory outbreak observed in our pigs was characterized by a low levels of acute phase proteins and high levels of adaptive immune markers that could be explained by a long-term persistent form (61). The humoral immune response is thought to be a key part of the host's protection against *App* (62), which also agree with the intense adaptive immune response observed in our study. Moreover, a high levels of acute phase proteins would be expected 4–5 days before the development of specific antibodies (63). Therefore, the low levels observed agree with a chronic state of the disease. The beneficial effect of enriched environments on the outcome of respiratory infections has been recently published, including *App*, which supported a role of the HPA axis on susceptibility to disease (64). Accordingly, we have found a large increase in salivary cortisol concentrations in pigs with dyspnea which could be interpreted as a stress condition. However, it was not possible to discern between a deficient welfare condition that increases the susceptibility to *App* or a poor welfare condition caused by the *App* infection.

In our study, a clear connection between immune and stress biomarkers has been pointed from an analytical point of view. However, this relation between immunity and stress status depends on the studied disease and support our initial hypothesis that each disease stimulates the distinct responses of the host in different magnitudes, and these differences could be used to discriminate between diseases.

The influence of stress on immunity and inflammation has been widely investigated. A cross-sensitization between stress and the immune system has been described in which pigs with a stronger HPA activation have a stronger proinflammatory cytokine response to *Escherichia coli* infection (65). However, the inflammatory response is modulated, following successive infections with lipopolysaccharide with a decreased amplitude of the response of salivary CRP and cortisol (66). It is widely believed that acute stress enhances immune function whereas chronic stress suppresses the response and increases susceptibility to disease (3).

Moreover, it has been reported that different stressors elicited different physiological stress responses in the pig (11), which corresponds to our results, in which, diseased pigs could show the increases in markers of HPA axis, autonomic nervous system, or both, depending on the pathological condition. Despite these variations in the level of activation of the different host responses, overall positive correlations were observed between psychological stress markers and immune markers as previously stated in experimental stress studies (11, 12).

The relation between oxidative stress and inflammatory reactions has also been studied in several infectious diseases in pigs such as pneumonia, enteritis, and sepsis (8). In this study, associations between salivary oxidative stress and immune markers were observed; however, those associations varied with the pathological condition analyzed. Additionally, oxidative stress could be generated by immune activation, physical exercise, or stress (67), and stressors can enhance oxidative stress reactions (7). Accordingly, in our study, we have also shown associations between the psychological stress and oxidative stress markers.

Nevertheless, a more practical purpose of the study was to monitor the behavior of all the selected biomarkers in the different pathological conditions to search for analytical patterns that could discriminate between disease conditions. Using the complete set of salivary biomarkers, our preliminary PCA analysis could differentiate all the pathological conditions studied from the group of healthy pigs. The distance between the group of pigs with lameness, rectal prolapse, and dyspnea from the healthy condition was evident. Nevertheless, distances between some diseases, specifically tail-biting lesions and diarrhea, should be higher for optimal clinical applications. Therefore, further studies with larger number of animals or even additional biomarkers should be performed to increase the proportion of variance explained by the analysis.

These preliminary results demonstrate a great potential for salivary analytics to be further exploited for disease characterization using machine learning methods. However, some additional studies should be performed to improve the diagnostic utility of the salivary clinical data in near future.

In conclusion, an analytical connection between immune system and stress has been identified with variations between porcine diseases. The study of the behavior of a panel of biomarkers of innate and adaptive immune response and psychological and oxidative stress status have allowed us to have a first porcine disease differentiation approach. At a practical level, the findings should be explored in a larger number of animals for successfully discriminating between pathological conditions in pigs using salivary analytics. This approach could be used to monitor health and welfare in pig production for the early diagnosis and the prevention of welfare and health problems.

## Data Availability Statement

The datasets presented in this study can be found in online repositories. The names of the repository/repositories and accession number(s) can be found below: http://hdl.handle.net/10201/117192.

## Ethics Statement

The animal study was reviewed and approved by Ethics Committee of the University of Murcia. Written informed consent for participation was not obtained from the owners because farm owners were appropriately informed about experimental procedures prior their implementation by the official veterinary.

## Author Contributions

JSo and AG contributed to conception and design of the study. AG and MM performed the laboratory analysis. AG organized the database. FI-L and IH performed the statistical analysis. JSá wrote the first draft of the manuscript. MM wrote the sections of the manuscript. All authors contributed to the manuscript revision, read, and approved the submitted version.

## Funding

The study was supported by the grant PID2020-116310RB-I00 and funded by MCIN/AEI/10.13039/501100011033.

## Conflict of Interest

JSá was employed by the company Cefu SA. The remaining authors declare that the research was conducted in the absence of any commercial or financial relationships that could be construed as a potential conflict of interest.

## Publisher's Note

All claims expressed in this article are solely those of the authors and do not necessarily represent those of their affiliated organizations, or those of the publisher, the editors and the reviewers. Any product that may be evaluated in this article, or claim that may be made by its manufacturer, is not guaranteed or endorsed by the publisher.
