# Supplementary material for: The Connection Between Stress and Immune Status in Pigs: A First Salivary Analytical Panel for Disease Differentiation
Source: Front Vet Sci. 2022 Jun 16;9:881435. doi: 10.3389/fvets.2022.881435 (PMC9244398; doi:10.3389/fvets.2022.881435)
Supplement: Supplementary file 1 [file Table_1.DOCX]

**Supplementary table 1.** Statistical test used for the comparison between animals suffering from a pathological condition (tail biting n = 13, rectal prolapse n = 13, diarrhea n = 13, lameness n = 14, or dyspnea n = 14) and healthy animals (n = 10) from the same farm and the p value for the comparison. For more information about size effect see table 1 in the main text. Abbreviations: CRP = C-reactive protein; Hp = Haptoglobin; ADA = Adenosine deaminase; TAC = Total antioxidant capacity; TOS = Total oxidant status; TP = Total protein.

| Symptoms | Variable | Test | p-value |
| --- | --- | --- | --- |
| Tail biting | CRP | t-test with Welch’s correction | 0.0285 |
|  | Hp | t-test with Welch’s correction | 0.0083 |
|  | ADA | Mann Whitney test | 0.0422 |
|  | Cu | Mann Whitney test | 1.0000 |
|  | Zn | Mann Whitney test | 0.5116 |
|  | PT | t-test with Welch’s correction | 0.2664 |
|  | TAC | t-test with Welch’s correction | 0.1174 |
|  | TOS | t-test | 0.5608 |
|  | Ratio TOS/TAC | Mann Whitney test | 0.9705 |
|  | Amylase | t-test with Welch’s correction | 0.0296 |
|  | Cortisol | Mann Whitney test | 0.0411 |
| Prolapse | CRP | t-test with Welch’s correction | 0.0017 |
|  | Hp | t-test with Welch’s correction | 0.0018 |
|  | ADA | Mann Whitney test | 0.0052 |
|  | Cu | t-test with Welch’s correction | 0.0026 |
|  | Zn | t-test with Welch’s correction | 0.0066 |
|  | PT | t-test | 0.0004 |
|  | TAC | Mann Whitney test | 0.1862 |
|  | TOS | t-test | 0.0565 |
|  | Ratio TOS/TAC | Mann Whitney test | 0.0653 |
|  | Amylase | Mann Whitney test | 0.8917 |
|  | Cortisol | t-test with Welch’s correction | 0.0139 |
| Diarrhea | CRP | Mann Whitney test | 0.0002 |
|  | Hp | Mann Whitney test | 0.0214 |
|  | ADA | t-test | 0.2578 |
|  | Cu | t-test | 0.0707 |
|  | Zn | Mann Whitney test | 0.6482 |
|  | PT | t-test | 0.1555 |
|  | TAC | t-test | 0.6370 |
|  | TOS | t-test | 0.6104 |
|  | Ratio TOS/TAC | Mann Whitney test | 0.3704 |
|  | Amylase | t-test with Welch’s correction | 0.0206 |
|  | Cortisol | Mann Whitney test | 0.0342 |
| Lameness | CRP | t-test with Welch’s correction | 0.0763 |
|  | Hp | t-test with Welch’s correction | 0.0027 |
|  | ADA | Mann Whitney test | 0.0100 |
|  | Cu | Mann Whitney test | 0.0155 |
|  | Zn | Mann Whitney test | 0.1375 |
|  | PT | t-test with Welch’s correction | 0.0091 |
|  | TAC | Mann Whitney test | 0.2591 |
|  | TOS | Mann Whitney test | 0.1375 |
|  | Ratio TOS/TAC | t-test with Welch’s correction | 0.2926 |
|  | Amylase | t-test with Welch’s correction | 0.0001 |
|  | Cortisol | Mann Whitney test | 0.0643 |
| Dyspnea | CRP | Mann Whitney test | 0.4031 |
|  | Hp | Mann Whitney test | 0.0828 |
|  | ADA | t-test with Welch’s correction | 0.0000 |
|  | Cu | t-test with Welch’s correction | 0.0000 |
|  | Zn | t-test with Welch’s correction | 0.0001 |
|  | PT | t-test | 0.1674 |
|  | TAC | t-test | 0.2747 |
|  | TOS | t-test | 0.0097 |
|  | Ratio TOS/TAC | Mann Whitney test | 0.0559 |
|  | Amylase | Mann Whitney test | 0.0821 |
|  | Cortisol | t-test with Welch’s correction | 0.0025 |
