# Supplementary figures and images for: The Connection Between Stress and Immune Status in Pigs: A First Salivary Analytical Panel for Disease Differentiation
Source: Front Vet Sci. 2022 Jun 16;9:881435. doi: 10.3389/fvets.2022.881435 (PMC9244398; doi:10.3389/fvets.2022.881435)

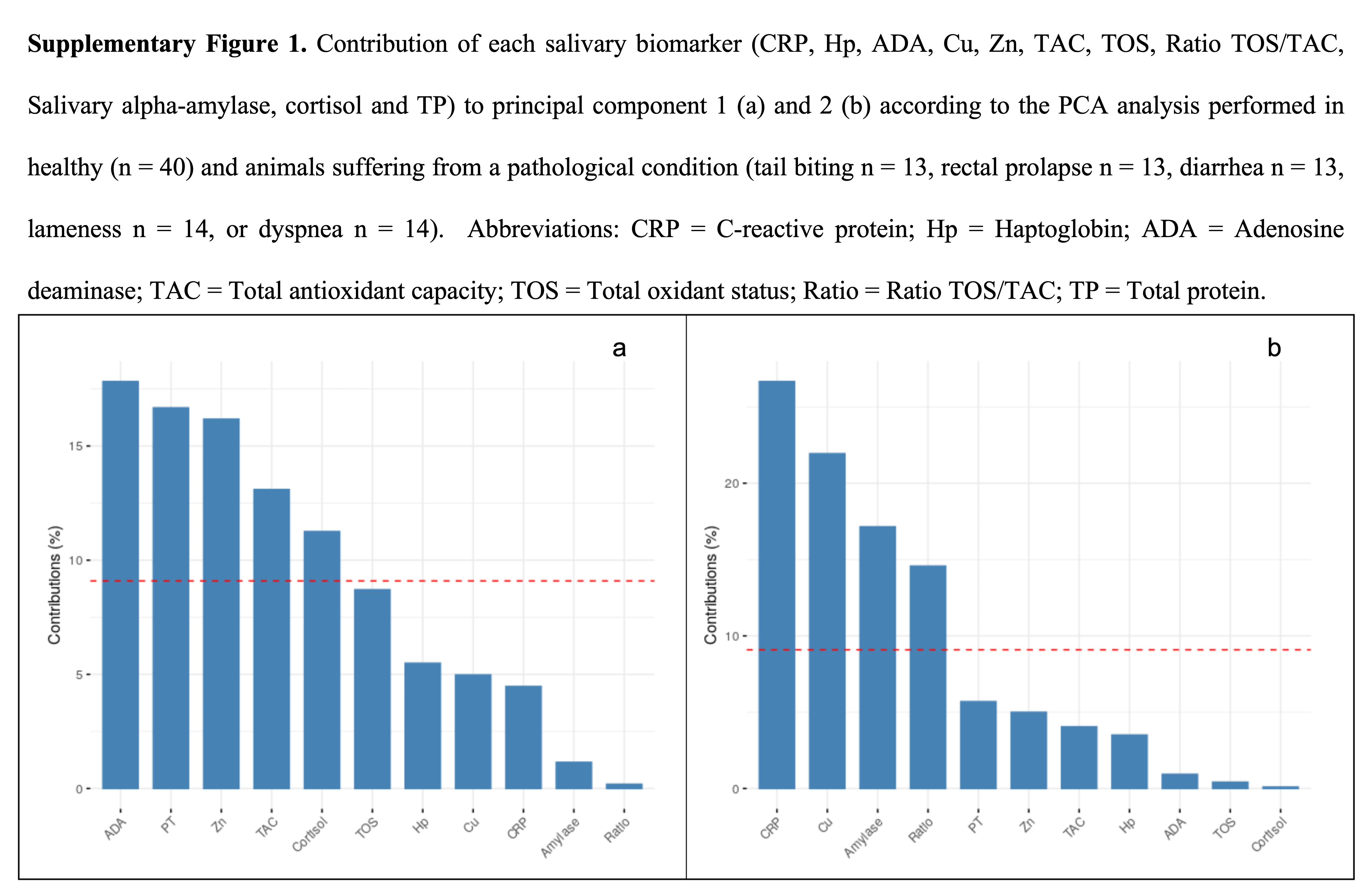

Supplement: Supplementary file 2 [file Image_1.TIFF]
